# The Mindful Self: A Mindfulness-Enlightened Self-view

**DOI:** 10.3389/fpsyg.2017.01752

**Published:** 2017-10-13

**Authors:** Qianguo Xiao, Caizhen Yue, Weijie He, Jia-yuan Yu

**Affiliations:** ^1^Laboratory of Emotion and Mental Health, Chongqing University of Arts and Sciences, Chongqing, China; ^2^School of Education, Inner Mongolia Normal University, Hohhot, China; ^3^School of Psychology, Nanjing Normal University, Nanjing, China

**Keywords:** mindfulness, self, self-development, self-view, self-attitude, Buddhist psychology

## Abstract

This paper analyzes studies of mindfulness and the self, with the aim of deepening our understanding of the potential benefits of mindfulness and meditation for mental health and well-being. Our review of empirical research reveals that positive changes in attitudes toward the self and others as a result of mindfulness-enabled practices can play an important role in modulating many mental and physical health problems. Accordingly, we introduce a new concept—the “mindful self”—and compare it with related psychological constructs to describe the positive changes in self-attitude associated with mindfulness meditation practices or interventions. The mindful self is conceptualized as a mindfulness-enlightened self-view and attitude developed by internalizing and integrating the essence of Buddhist psychology into one’s self-system. We further posit that the mindful self will be an important intermediary between mindfulness intervention and mental health problems, and an important moderator in promoting well-being. More generally, we suggest that the mindful self may also be an applicable concept with which to describe and predict the higher level of self-development of those who grow up in the culture of Buddhism or regularly engage in meditation over a long period of time.

## Introduction

Buddhist psychology is largely focused on analyzing and understanding the nature of the self, and many positive effects of meditative practice based on Buddhist psychology have been documented by researchers interested in self-processes (Gallagher and Shear, 1999). The central tenet of Buddhist psychology with respect to the self is that of “no self,” which posits that there is no unchangeable self. This differs from some schools of Western psychology in considering the concept of the self, though in some descriptions there are many similarities (McIntosh, 1997). In modern Western psychology, the self is constructed as a definable knowable entity with particular characteristics, universal needs, and somewhat predictable developmental thrusts (Sedikides and Spencer, 2007; Chan, 2008; Donner, 2010). For most people, the self refers to the things immediately connected to their body, and more importantly to their mind. It seems self-evident that perceptually, “I am the mind and the mind is I” (Chan, 2008). Social-cognitive approaches to the self, as well as clinical practice, teach people that it is important to expend much energy solidifying and bolstering their sense of self, (re)-establishing secure attachments, as well as promoting self-esteem. However, Buddhist teachings maintain that the self is an illusion that is truly the cause of much of our suffering. Thus, according to Buddhism, people should let go of as many attachments as possible, which are very difficult to give up in actuality. As a result, there appears to be a fundamental paradox in current clinical practice. It is undeniably important to develop continuity, identity, and an ongoing sense of self for all who suffer from pathological disturbances in their subjective sense of selfhood; yet, under Buddhist psychology, it is equally clear that adhering to a sense of personal continuity and self-identity results in chronic discontent and psychic conflict (Engler, 1984; Rubin, 2013). In effect, neither theoretical position should be favored over the other: overemphasizing the approach of Buddhist psychology may bring about an impairment of an individual’s self-conception, while focusing disproportionately on self-entity could lead to the formation and fantasy of self-stability (Rubin, 1996). A cogent theory of self should take into consideration aspects from both of these two standpoints, helping individuals to be more flexible (Falkenström, 2003).

Over the past two decades, an increasing number of researchers in the West have recognized that Buddhist doctrines (especially those concerning meditation practice), as well as the Chan (Zen) form of Chinese Buddhism, offer profound observations and effective methods with which to enlighten the experiencing self (Falkenström, 2003). Numerous studies have suggested, for example, that mindfulness meditation offers significant positive effects in reducing various physical and mental symptoms, enhancing self-functioning (Rigby et al., 2014), facilitating self-integration (Rubin, 2013), and altering the perspective of self-observation (Shapiro et al., 2006). The present study further contends that, consistent with the traditional aims of meditation in Eastern cultures, where it has been practiced for 1000s of years, the principal cause or mechanism underlying all these positive changes of self attained through Western scientific mindfulness practice or intervention is the reconstruction of a mindful self-view and attitude. In other words, we argue that positive changes such as these result from the integration and internalization of the mindful or meditative self-view and attitude which encompass notions of the non-self, impermanence, non-attachment, and equanimity. Therefore, we propose a new concept—the “mindful self”—to describe the mindfulness-enlightened self-view and attitude as these are explored in the present study. The paper describes the connotations of this concept and its psychological functioning as a new self-construct in the context of adult self-development.

## Mindfulness in Buddhism and Psychology

Buddhist psychology is an in-depth examination of the self that aims to lead humans to a flourishing life, while mindfulness meditation is a central factor in helping one to live such a life (Edge, 2014). Buddhist psychology affirms that there is no such thing as a permanent, unchanging self (Olendzki, 2010), and further contends that suffering pervades human existence and is chiefly caused by one’s greed, hatred, and delusion concerning what is felt and seen, as well as an illusory belief in the notion that there is an independent, permanent self. According to the Buddha, the only way to eradicate human anguish or suffering is to remove the attachment (*upadana*) or craving (*trsna*) of our mind toward various things or concepts to which we are attached. Mindfulness meditation is one of the most important elements of the Buddha’s “noble eightfold path” to end suffering and instill wisdom. Principally, mindfulness in Buddhist teaching is viewed as a fundamental pathway through which to become aware of the causes and sources of suffering and to attain enlightenment or an awakening, thereby enabling the individual to be less egoistical and obtain insight into the state of “no self.” According to Buddhism and its Chan School, when an individual has truly acquired complete enlightenment or insight into the “non-self,” they will have achieved full freedom of the mind.

The Buddhist concept “*sati*” was first translated as “mindfulness” from the Pali word by a British scholar of the language, Thomas William Rhys Davids, in 1881, based on his understanding of the *Mahasatipatthana Sutta*, which stresses how its practice is to watch how things “come to be” and how they “pass away” (Gethin, 2011). “Mindfulness meditation” has been studied extensively over the past two decades in the West, and is understood either as a process of self-observation (e.g., Martin, 1997), as a set of skills for self-regulation (e.g., Hayes and Wilson, 2003), or as a disposition or kind of cognitive ability (e.g., Kudesia and Nyima, 2014). Although there is some confusion regarding the differing definitions of mindfulness in terms of awareness, attention, and attitude (Harrington and Pickles, 2009; Mikulas, 2011), a widely adopted description is that it is a particular way of paying attention, a process of non-judgmental awareness (Goldstein, 2002; Bishop et al., 2004; Kabat-Zinn, 2005; Kabat-Zinn and Hanh, 2009), and an attitude of openness and acceptance (Shapiro et al., 2006). As an essential agent of a functioning mindfulness, mindfulness meditation is a way of looking deeply into oneself in a spirit of self-inquiry and self-understanding (Kabat-Zinn and Hanh, 2009) by a process of dis-identification or decentering with respect to the contents of the mind, and an experiential movement into a broader domain of consciousness which can make us aware of what we really are beneath the image of the ego (Gunaratana and Gunaratana, 2011). This detached awareness reduces an individual’s clinging to the contents of their mind that are associated with the person as themselves. Such a shift in perspective is called “re-perceiving” by Shapiro et al. (2006), who found that this is predominantly how mindfulness works in therapy. Ultimately, the mindfulness of both Buddhism and psychology is viewed as an important way to understand the nature of the self, and to obtain spiritual well-being.

## Mindfulness and the Self

In modern Western society, mindfulness is practiced, cultivated, and applied in far more diverse contexts than in the East, involving self-exploration, self-experience, and self-transformation (Schmidt, 2011). Correspondingly, there are large numbers of studies concerning mindfulness and the self, linking it to self-compassion (Neff K.D., 2003), self-acceptance (Carson and Langer, 2006; Dryden and Still, 2006), self-perspective change (Hölzel et al., 2011), self-consciousness (Evans et al., 2009; Ghorbani et al., 2010), self-concept (Crescentini and Capurso, 2015), self-deconstruction and reconstruction (Dahl et al., 2015), self-referential processing (Farb et al., 2007; Tang et al., 2015; Hadash et al., 2016), and so on. Growing numbers of studies and reports of clinical practice have suggested that mindfulness meditation practices bring about positive significant effects in reducing physical and psychological symptoms, and in improving active growth and well-being, as well as changing self-knowledge and the mode of self-referential processing. In this paper, we argue that the practices of mindfulness meditation lead to positive changes in the social-psychological functioning of the self at both a quantitative and a qualitative level, and that these quantitative and qualitative changes interact.

On the one hand, it has been suggested that engaging in mindfulness meditation practices or interventions (especially over long periods of time) is closely associated with increases in positive self-attitudes, such as non-attachment (or acceptance) (Monteromarin et al., 2016), self-compassion (i.e., self-kindness, not self-criticism) (Hollis-Walker and Colosimo, 2011), equanimity (not indifference) (Desbordes et al., 2015), and with becoming more compassionate to the self and equally so to others. In a certain sense, some of the above positive changes in self-attitude can also be observed or described at a qualitative level. For example, it has been suggested that compassion-focused meditation can facilitate an individual’s self-kindness or sense of warmth for those people with high levels of shame and self-criticism (Hofmann et al., 2011; Gilbert, 2014), making more shamed or self-critical people become more self-kind.

Moreover, it has been suggested that mindfulness is associated with decreasing self-identification with self-images (Graham et al., 2009; Edwards, 2013), moderating the defensive tendencies associated with lower ego-involvement (e.g., Kernis and Goldman, 2006; Weinstein et al., 2009). Consequently, it promotes self-knowledge; i.e., the knowledge of the nature of the self and the relation between the self and the content of experience (Jankowski and Holas, 2014). Further, it increases one’s ability to start experiencing a sense of the self as a transitory, interdependent, dynamic change process, rather than as a constant and unchanging entity. That is to say, it gradually changes implicit self-concepts and perspectives on the self at a qualitative level (Dambrun and Ricard, 2011; Hölzel et al., 2011), according to which self-referential processing becomes diminished, while first-person experiencing becomes enhanced (Hölzel et al., 2011). Instead of identification with a static self, there emerges a tendency to identify with the phenomenon of “experiencing” the self without psychological defense mechanisms.

In addition, mindfulness can change the mode of self-focused attention (Jain et al., 2007; Heeren and Philippot, 2011; Campbell et al., 2012). It has been suggested that mindfulness meditation makes one to be fully but impartially aware, and attentive to what is occurring without judgment, investment, or antipathy for what appears, which leads to clarity and accuracy in people’s perceptions and judgments (McIntosh, 1997; Ryan and Rigby, 2015). Within this enhanced clarity, the process of a repeatedly arising sense of self becomes observable to the meditator and facilitates a detachment from identification with the static sense of self, which has been termed “decentering” or “reperceiving” (Shapiro et al., 2006; Carmody et al., 2009). It has been postulated that paying attention and awareness to the transitory nature of this sense of self leads to the “positive deconstruction of the self” (Epstein, 1988).

The shift in self-awareness is viewed as one of the major active mechanisms of the beneficial effects of mindfulness meditation (Tang et al., 2015). The non-self-referential process of the present-moment experience is also seen as a unifying metacognitive process underlying a number of candidate mindfulness mechanisms characterized by dis-identification from internal experience, such as decentering, metacognitive awareness, and re-perceiving (Bernstein et al., 2015; Hadash et al., 2016). Following up on such studies, Vago and David (2012) proposed an “S-ART” framework (self-awareness, self-regulation, and self-transcendence) for understanding mindfulness, focusing on self-processing and the underlying neural systems, and based on existing neurocognitive research and empirical studies of mindfulness and the self. The S-ART framework uses self-processing to illustrate the complexity of the mechanisms of mindfulness that function to reduce suffering and create a sustainably healthy mind. Within this framework, mindfulness is described as reducing a distorted or biased sense of self and one’s relation to others and the external world through specific forms of mindfulness practices that develop a meta-awareness of self (self-awareness), an ability to effectively manage or alter one’s responses and impulses (self-regulation), and the development of a positive relationship between the self and others that transcends self-focused needs and increases prosocial characteristics (self-transcendence). Overall, S-ART sets out to show that mindfulness meditation practice is an effective and complex process that reduces suffering and creates a sustainably healthy mind in individual subjects.

In all, these findings suggest that mindfulness practices moderate implicit self-concepts and perspectives on the self (Levesque and Brown, 2007), and encourage positive functions of the self with a shift toward more healthy profiles (Olendzki, 2006; Hölzel et al., 2011; Crescentini and Capurso, 2015).

## The Definition and Connotations of the Mindful Self

The above studies suggest that mindfulness works beneficially on changing self-experiencing and self-understanding, softening one’s “centered” and “substantialized” self and, instead, internalizing and integrating the essence of Buddhist psychology (e.g., the non-self, and impermanence) into one’s self-system or self-concept. To describe such a changed and mindful self, which underlies mindfulness practices as well as dispositional mindfulness, we propose a new concept: the “mindful self (MS).” Further to a series of empirical and theoretical studies of mindfulness and the self, we define MS as a mindfulness-enlightened self-view that involves an embodied integration and internalization of the essence of the non-self in the process of mindfulness meditation practices or intervention. Here, being mindful means becoming aware or paying attention to everything. It means letting nothing occur without one’s conscious being aware of it (McIntosh, 1997; Rosenbaum, 2009), while “self-view” refers to self-understanding and self-attitude.

From the perspective of self-development, the connotations of the MS concept share some associations with terms such as “self-actualization” (Roger, 1951; Maslow, 1970) and “ego development” (Loevinger, 1977). It also shares some meanings with the higher level identified in Dabrowski’s (1964) theory of positive disintegration. The main connotations of self-actualization, ego-development, positive disintegration theory, and the mindful self (positive effects or changes of the self that are based on mindfulness meditation), are presented in **Table 1**. However, there are also substantial differences between the MS and self-actualization, ego-development, the theory of positive disintegration, as well as with dispositional mindfulness and “the quiet ego” (Bauer and Wayment, 2008), as discussed in the following sections.

**Table 1:** The main connotations of MS, self-actualization, ego development, and the positive disintegration theory.

Terms	The definition and the connotation description/Generalization
MS (mindful self)	**Definition:** A mindfulness-enlightened self-view and attitude, which takes the self as a process with awareness. **Connotation description:** **Mindfulness:** Here and now; Concentration; Non-judgmental (non self -critical), Self-awareness (Kabat-Zinn, 2003; Baer et al., 2006). **Selfless reference process**: Self-observation; Decentering, Metacognitive awareness, Re-perceiving (Han et al., 2010; Hölzel et al., 2011; Bernstein et al., 2015; Hadash et al., 2016); Self-de(re)construction (Epstein, 1988; Williams and Kabat-Zinn, 2011); Self-flexibility (Hayes et al., 2006; Ie et al., 2013; Atkins et al., 2015). **Self attitude:** Openness (Baer et al., 2006); Self-acceptance (Carson and Langer, 2006; Dryden and Still, 2006) Self-compassion/kindness (Neff K., 2003); Wisdom and Compassion (Hosking, 2007; Germer and Siegel, 2012); Non-attachment (Sahdra et al., 2010; Xuan et al., 2016); Equanimity (Lomas et al., 2015).
Self-actualization (Roger, 1951; Maslow, 1970)	**Definition:** A process through which one’s potential is developed in congruence with one’s self-perception and one’s experience (Leclerc et al., 1998). **Connotation description:** **Openness to experience:** Self awareness; Self trust; Self insight; Self acceptance; Openness; Empathy; Concentration; Here and now, Spontaneous; Intimate contact; Meaning to life; Commitment. **Reference to self:** Self responsibility; Positive self-esteem; Behave authentic and free; Acceptance of choices and actions/ consequences; Ethics (abstract from the Table 3 in Leclerc et al., 1998).
The higher stage of ego development (Loevinger, 1977)	**Definition**: Ego development is viewed as a process of maturation that involves transformations in the person’s self (Bailey, 2011). **Connotation/characterization:** Individuality; Self-awareness; Tolerance; Self-fulfillment; Wholeness; Conscientious; Complexity; Autonomy.
The higher level of positive disintegration theory (Dabrowski, 1964)	**Basic hypothesis:** The positive disintegration theory postulates human development is from positive disintegration of multilevel inner conflicts. **Connotation/characterization:** Level IV: Subject-object in oneself; Responsibility; Empathy; Self-control; Autonomy; Freedom from lower level drives and motivations. Level V: Life inspired by a powerful ideal such as: Equal rights, World peace, Universal love, Compassion, Sovereignty of all nations. Ultimate goal of development, Essence of one’s being; Creative instinct, Empathy, Identification with higher levels and personality ideal. (cited from Bailey, 2011).

### The Mindful Self and Self-actualization: The Authentic or True Self

The MS and self-actualization share some concepts, both emphasizing the importance of openness to experience, and incorporating ideas about the “here and now” and awareness. Beyond that, though, some constituents of mindfulness or Buddhist psychology are integrated into the MS, such as non-judgment, focusing on the present, non-attachment, and equanimity. An additional difference between the MS and self-actualization is in their respective underlying modes of the process of self-reference: the MS emphasizes a selfless reference process, whereas self-actualization prioritizes the self-reference process. In the Buddhist tradition, experiential self-reference is viewed as a cause of suffering, and the selfless process is seen as a key mechanism, achieved through mindfulness, with which to alleviate suffering (Olendzki, 2006). The purpose of mindfulness is essentially to help people become less self-centered (Rosenbaum, 2009) and to assist them in achieving cognitive transformations and obtain wisdom (Purser and Milillo, 2014), ultimately culminating in an undistorted vision of the self. Thus the MS weakens the self-reference process and the position of self-identification by underlining the importance of self-flexibility, decentering, non-attachment, and equanimity; in contrast, self-actualization strengthens the value of self-identification and positive self-esteem, which can be seen from the connotation description in **Table 1**. Prior studies have found evidence of some negative effects of pursuing high self-esteem and overly high self-identification. For instance, research has suggested that those with high self-esteem can be angry and aggressive toward others (Baumeister et al., 1996) and become more vulnerable to depression and reduced self-conceptual clarity (Kernis, 2005). High self-esteem has also been associated with self-enhancement bias (Sedikides and Gregg, 2008) and a high level of narcissism (Twenge and Campbell, 2009). On the other hand, evidence of the more positive effects of some Buddhist concepts is increasingly available. For example, it has been suggested that both self-compassion and mindfulness are more effective ways of helping individuals to develop a state of well-being than high self-esteem, with less ego-defensiveness and self-enhancement (Neff, 2011; Ostafin et al., 2015).

In addition, mindfulness is closely related to authenticity (Heppner and Kernis, 2007), which is also emphasized by self-actualization. It has been suggested that mindfulness enables an increase in “authentic functioning”— i.e., being aware of and regulating oneself (Avolio and Gardner, 2005)— and can help individuals to become more aware of one’s “true self” (Brown and Ryan, 2003; Leroy et al., 2013). However, there are some essential differences between the mindful self and the true self, though they share some common features such as emphasizing the importance of awareness, unbiased or non-judgmental processing, and authentic behavior (Kernis and Goldman, 2006). Researchers interested in the true self focus on the “psychological reality” of the true self and the consequences of the “true self” concept in everyday life (Schlegel and Hicks, 2011), in which the true self is considered as a state or trait. That is to say, the true self is an entity-oriented construct. As a matter of fact, people tend to believe that the true self is discovered “within” and tend to agree with statements that the true self is “something very basic about them” and that “it can’t really be changed,” even after writing about a significant change they have observed in either themselves or a close friend (Schlegel and Hicks, 2011). Correspondingly, the mindful self is a new construct based on the internalization and integration of the doctrine of “no-self,” taking the self “as a process,” which can be seen as a process of “de-entity” of the self; i.e., a process of attenuating self-entity.

### The Mindful Self, Ego Development, and the Positive Disintegration Theory

The MS is also different from ego development and the positive disintegration theory. “Ego development” is conceptualized as a mature trait characterized by five implicit features: individuality, self-awareness, complexity, wholeness, and autonomy. Ego development attempts to describe the developmental stages in the ways in which individuals make meaning of their personal life experiences and the world at large (Bailey, 2011). However, it lacks a clear distinction between content and form as well as any logical nature to the sequence of levels (Broughton and Zahaykevich, 1988). The “theory of positive disintegration” describes an important underlying mechanism concerning mental development by highlighting the process of a positive disintegration of tension, inner conflict, and anxiety from lower to higher levels of mental life, thereby emphasizing the importance of emotion in adult self-development. Through the process of positive disintegration, adolescents gain the capacity to differentiate and then to integrate their own distinct inner experiences (Laycraft, 2011). As **Table 1** shows, the MS is associated with the higher levels of both ego development and the positive disintegration theory. All three concepts are closely related to self-development but from different perspectives, and the MS may represent a particular self-view underlying the higher levels of ego development and the positive disintegration theory, especially for those who grow up in a Buddhist culture and those who practice mindfulness or Chan meditation over a long period.

### The Mindful Self and the Quiet Ego

The MS is intrinsically different from what is described as the “quiet ego.” In their study of how people transcend egotism, Bauer and Wayment (2008) identified the quiet ego as a middle ground within which to seek a balanced and integrated self-identity between the self and others. It is construed as being not excessively self-focused but also not markedly other-focused; that is, as an identity that incorporates others without losing the self. The authors state that the quiet ego, as a balanced self-identity, encompasses four principal features: (1) detached awareness; (2) interdependence; (3) compassion, involving acceptance, empathy, and a desire for the cultivation of happiness; and (4) growth, including a consideration of the development of the self and others (Bauer and Wayment, 2008). Obviously, this concept emphasizes the balance between the self, others, and growth. However, it lacks a tangible “growing-up feature.” The authors argue that, if an ego becomes excessively quiet, it can lose its identity or be quashed (Bauer and Wayment, 2008). Consequently, it is difficult to comprehend how much “quiet” is optimal or preferable. By contrast, the MS is constructed as a positive self-attitude, a state-like quality emphasizing the continuous process of self-awareness, self-insight, and integration within a lifespan perspective of recognizing that a real self is not a stationary thing but a process of formation (Rogers, 1961). The more an individual has a higher integration and internalization of mindfulness, the more mature, free, and flexible they will be in mind and behaviors. This is consistent with ideas featured in the ancient Chinese text the *Zhuangzi* (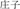)—such a self would be embodied, would be fully open to each new experience, and would respond in an appropriate and harmonious way, fitting in effortlessly (Berkson, 2005).

### The Mindful Self and Dispositional Mindfulness

“Dispositional mindfulness” refers to an individual’s capacity and tendency to abide in mindful states over time (Brown et al., 2007), comprising continued well-developed attention and inhibitory control (Stillman et al., 2014). It is conceptualized as a trait (Goodall et al., 2012) or as a mindful personality (Hanley, 2016). Operationally, the factors in Baer et al.’s (2006) Five Facet Mindfulness Questionnaire—observing, describing, acting with awareness, non-reaction to inner experience, and non-judgment of inner experience—are commonly taken to measure an individual’s dispositional mindfulness (Baer et al., 2006). Many studies suggest that a higher dispositional mindfulness benefits a wide range of psychological and social outcomes, including an increase in life satisfaction, more positive affects, less negative affects (Brown and Ryan, 2003), and an increase in self-esteem (Pepping et al., 2013). It has also been found that dispositional mindfulness is significantly correlated with each factor in the Five Factor Model of personality (Hanley, 2016).

Clearly, the MS and dispositional mindfulness are two interrelated but different concepts. As summarized above (see also **Table 1**), mindfulness practices can bring about three aspects of positive change: cognitive; behavioral (both of which are principally associated with dispositional mindfulness); and self-view and attitude (which are principally associated with the MS). Although the Five Facet Mindfulness Questionnaire includes attitudes such as acceptance and openness, it does not capture the mindful attitude directly. Rather, the MS can be seen as a “result” of mindfulness practice or as the attitudinal aspects of those who have a high trait of dispositional mindfulness. A number of studies have suggested that a positive change of attitude toward the self and others plays an important role in the psychological effects of mindfulness (Adair, 2013; Xu et al., 2016; Yang and Mak, 2016). If the process of therapies based on mindfulness does not foster positive attitudes such as acceptance and equanimity, awareness itself may not contribute too much on psychological well-being (Cardaciotto et al., 2008). In addition, heightened awareness can even have adverse effects on patients with panic disorder and can even have a chronic negative affect on patients with panic disorder (Mor and Winquist, 2002). Therefore, it is necessary to distinguish the two aspects—behavioral tendency and attitude—in connection with mindfulness. Such separation is not only favorable with respect to studying the underlying mechanism or the relationship between mindfulness and psychological health; it is also conducive to psychological intervention and education. Studying the underlying change of attitudes behind mindfulness is of obvious value here, and the MS is a meaningful step in this undertaking.

Given the considerable differences between the MS and related concepts, we argue that it is necessary, theoretically, to propose a new concept with which to describe the positive changes to the self that are associated with mindfulness meditation practices or interventions. As a definition, the MS is conceptualized as a mindfulness-enlightened self-view and attitude attained via a process of internalizing and integrating some thoughts from Buddhist psychology into one’s self-system, summarized as follows. First, the MS acknowledges that human beings have many basic desires and needs, materially and spiritually, because nobody can be without needs, desires, or discrimination in their worldly life. But, secondly, the MS emphasizes that we should not over-identify or become overly attached to one’s various desires and needs, no matter how positive or negative they are. Thirdly, the MS lays weight on understanding the dependent, impermanent essence of the self, viewing it as a flow process, or simply as a mental phenomenon rather than as an entity, which seems to be some of the important elements involved in becoming a person (Rogers, 1961). That is, a mindful person no longer treats the content of experience as objective facts and as a direct read-out of reality (Jankowski and Holas, 2014). Fourthly, the MS emphasizes the significance of elaborated self-awareness and self-flexibility, and the attitude of an “ordinary mind” in promoting the positive function of the self and maintaining mental health. Thus, fifthly, the MS encourages people to change their perspective on the self, viewing the self as a process instead of an “entity” or product of reification, in which mindfulness constitutes a facilitator for “I-self” over “me-self” experience as people grapple with their relative realities; it is a resource for organizing behavior by supporting the synthetic tendencies of self-as-process (Ryan and Rigby, 2015).

As far as the secular individual is concerned, we can not live in the absolute level of reality that has the characteristic or the true nature of the “empty self” or “no-self.” In our worldly lives, we can not live in the absolute level of non-dualism and indiscrimination. The aim of mindfulness practice for secular people is not to transcend the cycle of life and death that is proclaimed by Buddhism as a religion. Instead, the significance of internalizing and integrating the essence of Buddhist psychology organically into one’s self-system lies in enlightening the mind to wisdom such as non-attachment, let-going to alleviate our suffering, and coping with the uncertain challenges of life. We can be mindfully aware of our selves and experiences at the relative level, while simultaneously recognizing the absolute reality of phenomena (Gyatso, 2002). Presumably, in fact, mindfulness fully practiced will lead one along the “middle way” (Hanh, 1999)—i.e., seeking a path between the self and no-self. We conceptualize the aim here as the mindful self, which takes the self as a process with awareness. The more self-as-process is active, the more the person experiences his or her behaviors as volitional and autonomous, and the more his or her actions are experienced as wholehearted and authentic (Ryan and Rigby, 2015).

## The Ms and its Psychological Functioning

Based on the above analysis, we posit that the MS, as a mindfulness-edified self-view, is the primary cause or mechanism behind the stable positive changes attained through mindfulness meditation practices or interventions. There are several reasons to believe that the MS is a robust indicator in determining the significant positive effects and their relationships with mental health problems. On the one hand, the main aim of mindfulness is to cultivate a meditative attitude in order to gain a peace of mind based on the embodied insight of impermanence (*anicca*) or not-self (*anatta*) and suffering or pervasive “unsatisfactoriness” (*dukkha*) (Khong, 2009). As Khong (2009, p. 14) elucidates:

“This brings us to one of the most important teachings of the Buddha, that gaining insight into the ontological nature of reality can bring about a change of attitude at the individual level. The transformation of perspective involves adopting a meditative attitude of non-attachment, acceptance and letting be, and letting go. Without this change in attitude, understanding the *dukkha* [suffering] could become merely an intellectual exercise.”

On the other hand, according to the teachings of the Chinese Chan (Zen) school, the foremost means of liberating the mind is to treat “the lost/deluded self” directly. Chan Buddhism suggests that, provided the nature of the self has changed harmoniously and is fully enlightened; the psychological symptoms of mental problems will disappear or diminish as a matter of course. As the originator of the Chan school of Buddhism expounded after attaining enlightenment:

“The mind has the capacity for great things, and is not meant for practicing petty ways. Do not talk about emptiness with your mouth all day and in your mind fail to cultivate the conduct that you talk of …When you become enlightened in a sudden insight, you do not grasp onto the cultivation of external things. When your own mind constantly gives rise to right views, afflictions and defilement can never stain you. That is what is meant by seeing your own nature” (Hui-Neng, 2005).

Thus, traditional mindfulness meditation emphasizes the significance of changing attitudes and the most fundamental knowledge of oneself in the practice or intervention of mindfulness meditation. Correspondingly, attitudes such as non-attachment, equanimity, “not doing,” and self-compassion are the most important components of the MS. Several studies have found that these attitudes play an important role in modulating the effects of mindfulness and psychological health. For example, it has been shown that non-attachment substantially mediated the links between facets of mindfulness and the outcome variables of satisfaction with life and life effectiveness (Sahdra et al., 2015). Similarly, other studies have found that self-compassion moderated the relationship between self-stigma content and life satisfaction among people living with HIV (Yang and Mak, 2016). Moreover, a number of studies have suggested that expressing compassion, kindness, or love to oneself helps to reduce the tendency of hostility or anger to others (Analayo, 2004), increases positive affects and decreases negative affects (Hofmann et al., 2011), and decreases shame and self-criticism (Gilbert, 2009). Furthermore, some studies have discovered that self-compassion is a stronger predictor of well-being than mindfulness (Van Dam et al., 2011; Woodruff et al., 2014). In addition, equanimity, or an equal and receptive attitude toward all beings, is also an important concept that captures, potentially, a significant psychological element in the improvement of well-being (Desbordes et al., 2015). Therefore, we infer that the MS is an important intermediary between mindfulness intervention and mental health problems, and in promoting well-being.

In addition, and more generally, it would be of benefit to consider the MS as a particular standard of psychological maturity in the context of self-development. Psychological standards of maturity involve the development of favorable attitudes toward the self, others, and work-related motives, and of a behavior-guiding system of values (Greenberger and Sørensen, 1974). The positive changes based on mindfulness that are able to meet the criteria of psychological maturity, as summarized by Golovey et al. (2015), include qualities or traits of responsibility, reflexiveness, awareness, self-acceptance and self-respect, integrity and congruence, and so on. A number of studies have suggested that mindfulness meditation makes one more mature and leads to more autonomous behavior (Levesque and Brown, 2007; Ireland, 2013). It is certainly clear that mindfulness practice is associated with awareness and self-acceptance, but it has also been found that a higher level of mindfulness is connected to more self-congruence, reflected in a higher concordance between implicit (non-conscious) and explicit (conscious) assessments of self-related attributes (Thrash and Elliot, 2002; Brown and Ryan, 2003). Accordingly, and further to the comparison of the MS and self-actualization above, we submit the MS as a valid, useable concept in this context and a more appropriate indicator than self-actualization to describe and predict the higher level of self-development of those who grow up in the culture of Buddhism and those who engage in meditation in the long term.

## Conclusion

Extensive evidence exists to suggest that cultivating a mindful or meditative attitude toward oneself and others, which we have conceptualized as the MS, is of great benefit to one’s health and well-being. As a theoretical prediction based on a review of empirical research, we posit that the MS can also be an important intermediary between mindfulness intervention and mental health problems, and in promoting well-being. More generally, we propose that the MS is an applicable concept with which to describe and predict the higher level of self-development of those who grow up in a Buddhist culture and those who engage in a long-term practice of mindfulness meditation. However, further empirical study is required. In particular, the basic components of the MS, as well as its incremental and predictive validity, need to be constructed and examined by investigating the self-views and attitudes of those people who are deemed to possess a high level of mindfulness.

## Author Contributions

The design and writing of this paper was completed by QX. WH and CY took part in a discussion. J-yY was mainly engaged in the process of design, revision, and discussion.

## Conflict of Interest Statement

The authors declare that the research was conducted in the absence of any commercial or financial relationships that could be construed as a potential conflict of interest.
